# Testing CP-violation in a heavy Higgs sector at CLIC

**DOI:** 10.1140/epjc/s10052-025-14369-6

**Published:** 2025-06-25

**Authors:** Kingman Cheung, Ying-nan Mao, Stefano Moretti, Rui Zhang

**Affiliations:** 1 Department of Physics and CTC, National Tsing Hua University, Hsinchu, 300 Taiwan; 2 Division of Quantum Phases and Devices, School of Physics, Konkuk University, Seoul, 143-701 Republic of Korea; 3 Department of Physics, School of Physics and Mechanics, Wuhan University of Technology, Wuhan, 430070 Hubei China; 4 School of Physics and Astronomy, University of Southampton, Southampton, SO17 1BJ UK; 5 Department of Physics and Astronomy, Uppsala University, Box 516, 751 20 Uppsala, Sweden; 6 Theoretical Physics Division, Institute of High Energy Physics, Beijing, 100049 China; 7 Shanghai Key Laboratory of Deep Space E.ploration Technology, Shanghai Institute of Satellite Engineering, No.3666 Yuanjiang Road, Shanghai, 201109 China

## Abstract

We propose a method for probing CP-violation in the heavy (pseudo)scalar sector of an extended Higgs model, in which we make simultaneous use of the *HVV* ($V=W^\pm , Z$) and $Ht\bar{t}$ interactions of a heavy Higgs state *H*. The CP-even component of *H* can be probed through the tree-level *HVV* interaction, while the CP-odd component of *H* can be probed if the final $t\bar{t}$ pair can be tested to form a $^1S_0$ state. We can then confirm CP-violation if both CP-even and CP-odd components of *H* are discovered. This is possible at the Compact Linear Collider (CLIC) by exploiting *H* production from vector-boson fusion (VBF) and decay to $t\bar{t}$ pairs. We analyze the distribution of the azimuthal angle between the leptons coming from top and antitop quarks, which allows one to disentangle the CP nature of such a heavy Higgs state. We also show its implications for the two-Higgs-doublet model (2HDM) with CP-violation.

## Introduction

CP-violation was first discovered in the long-lived *K*-meson rare decay channel $K_L\rightarrow 2\pi$ in 1964 [1]. Additional CP-violation effects have since been measured in the *K*-, *D*- and *B*-meson sectors over the past several decades [2–4] (see [5] for a historical review). All these measured CP-violation effects are consistent with the explanation given through the Kobayashi–Maskawa (KM) mechanism [6], which represents another success of the Standard Model (SM) of particle physics. However, it is necessary to search for CP-violation sources beyond the SM (BSM). One important reason is that the amount of CP-violation contained in the SM is not enough to explain the matter–antimatter asymmetry in the Universe [7–9].

Theoretically, many BSM scenarios can accommodate additional CP-violation sources to remedy such a flaw of the SM. However, they are strongly constrained by experiments. Specifically, measurements of the electric dipole moments (EDMs) of, e.g., electrons and neutrons [10–12] have already set stringent limits on such new sources in efforts to reveal their existence [13–16], as the sensitivities involved are far above the SM predictions [17–19]. However, the EDM measurements, being very inclusive, are only an “indirect” probe of such new CP-violation sources, which means that even if we discovered CP-violation above the SM predictions, it is unlikely that we could determine the actual interactions involved. Conversely, collider experiments, despite having weaker sensitivities to CP-violation than EDM measurements, can afford one—thanks to the extensive variety of exclusive observables that one can define herein—with a “direct” probe of CP-violation.

The case for the complementarity of these two experimental settings can easily be made for BSM frameworks with extended Higgs sectors [20–24]. As an example, Ref. [25] studied both EDM and collider effects in a two-Higgs doublet model (2HDM) [23] with explicit CP-violation, in which nonzero EDMs are expected to be its first signal, with collider effects able to provide additional information.^1^

After the discovery of the 125 GeV Higgs boson at the Large Hadron Collider (LHC) [47–49], testing its CP properties is crucial to ascertaining the structure of the underlying Higgs sector. On one hand, current measurements are consistent with the CP-even (or scalar) state of the SM. On the other hand, an additional Higgs state, possibly mixing with it, may have different CP-properties (e.g., being pseudoscalar or a mixture of the two). To stay with 2HDMs, in these BSM scenarios, an effective method to test the CP-properties of the resulting physical states is trying to test CP-violation effects in the Yukawa interactions between such an additional (heavy) Higgs boson and fermions via the Lagrangian term

1 $$\begin{aligned} {\mathcal {L}}\supset -\bar{f}\left( g_S+\text {i}g_P\gamma ^5\right) fH. \end{aligned}$$

Generally, $f=t$ or $\tau$, because a top quark or $\tau$ lepton decays quickly enough so that the spin information of the decaying object is protected in its final-state distributions, which is useful for testing the CP-properties of a scalar *H*. In fact, the spin and CP quantum numbers correlate strongly in the Yukawa interaction. Phenomenologically, there are many works in the literature aimed at testing CP-violation in $Ht\bar{t}$ [25, 37–39, 50–65] or $H\tau ^+\tau ^-$ [27, 35, 40, 41, 66–73] interactions at colliders.

In addition, one can also probe CP-violation in the purely bosonic sector, through the interactions between a Higgs state and the SM massive gauge bosons. To exploit this approach, we again need such an additional Higgs state *H* (while the SM-like 125 GeV Higgs boson is denoted as *h*). The general effective interactions among *h*, *H*, and $V=W^{\pm },Z$ are (with $\theta _W$ being the weak mixing angle)^2^

2 $$\begin{aligned}  &   {\mathcal {L}}\supset \left( \frac{2m^2_W}{v}W^{+,\mu }W^-_{\mu }+\frac{m^2_Z}{v}Z^{\mu }Z_{\mu }\right) \left( c_hh+c_HH\right) \nonumber \\  &   \qquad +\frac{c_{hH}g}{2c_{\theta _W}}Z^{\mu }\left( h\partial _{\mu }H-H\partial _{\mu }h\right) . \end{aligned}$$

We already know that $c_h\ne 0$ through current LHC measurements. If both $c_H$ and $c_{hH}$ are nonzero, we will confirm CP-violation in the Higgs sector, because in such a case, *h* and *H* cannot be CP eigenstates at the same time, as was shown in [74–76].

In the SM, as hinted above, the only CP-violation source is the complex phase in the Cabibbo–Kobayashi–Maskawa (CKM) matrix [6], which means that if there exists new CP-violation in Higgs interactions, the Higgs sector of the SM must be extended. Consequently, it becomes attractive to search for CP-violation through the dynamics of additional Higgs states, as done recently for 2HDMs, both fundamental and composite, in Refs. [38–41, 77], which indeed exploited either $Ht{\bar{t}}$ or *HVV* couplings.

In this paper, we propose a method for testing CP-violation through such a heavy *H* state, as we consider its interactions with both massive fermions and gauge bosons simultaneously. The advantage of this approach is that the CP-even component in *H* is confirmed through *HVV* ($V=W^\pm ,Z$) interactions while the CP-odd component in *H* is confirmed through $Ht\bar{t}$ interaction. For this purpose, we consider a process in which the heavy Higgs state *H* is produced through vector boson fusion (VBF), i.e., $W^+W^-$- or *ZZ*-fusion, and decays into top (anti)quark pairs ($t{\bar{t}}$). As collider setup, we choose an electron–positron design, in preference to a hadronic one, because of the cleanliness of the described signature therein and among the various future options for the latter. We favor the compact linear collider (CLIC) design [78–86] because its $\sqrt{s}$ can reach ${\mathcal {O}}(\text {TeV})$, and hence is comparable to the LHC reach. In our analysis, we take the beam polarization setup following [80–82], which will be described in detail in Sect. 2.2.

The paper is organized as follows. We describe our method in Sect. 2 using a model-independent formulation, and also perform the simulation studies. In Sect. 3, as an illustration, we apply our method to the 2HDM with CP-violation. Finally, we conclude in Sect. 4.

## Model-independent studies

### Method

Assuming a heavy scalar *H* is discovered, and focusing in this paper on the CP properties of this particle, its effective interactions with massive gauge bosons and fermions can be written in general as

3 $$\begin{aligned}  &   {\mathcal {L}}\supset c_VH\left( \frac{2m^2_W}{v}W^{+,\mu }W^-_{\mu }+\frac{m^2_Z}{v}Z^{\mu }Z_{\mu }\right) \nonumber \\  &   \quad -\mathop {\sum }_f\frac{m_f}{v}H\bar{f}\left[ \text {Re}\left( c_f\right) +\text {i}\text {Im}\left( c_f\right) \gamma ^5\right] f. \end{aligned}$$

**Fig. 1:**
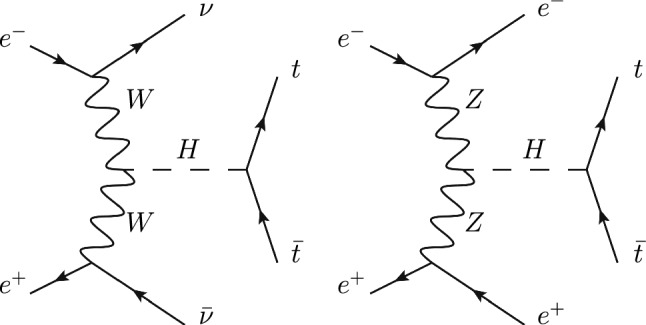
The Feynman diagrams for VBF processes $VV\rightarrow H\rightarrow t\bar{t}$, where $V=W^\pm ,Z$

We choose the VBF processes $VV\rightarrow H\rightarrow t\bar{t}$ where $V=W^\pm$ or *Z*, and the Feynman diagrams are shown in Fig. 1.

If such processes can be measured, we have $c_V\ne 0$, and thus the CP-even component of *H* will be confirmed. For the final-state $t\bar{t}$, if $\text {Im}\left( c_t\right) =0$ and $\text {Re}\left( c_t\right) \ne 0$, meaning a pure CP-even $Ht\bar{t}$ coupling, the $t\bar{t}$ pair will form in a $^3P_0$ state. Instead, if $\text {Re}\left( c_t\right) =0$ and $\text {Im}\left( c_t\right) \ne 0$, meaning a pure CP-odd $Ht\bar{t}$ coupling, the $t\bar{t}$ pair will form a $^1S_0$ state. In the CP-violation scenario, there will be both $^3P_0$ and $^1S_0$ types of $t\bar{t}$ final states. Thus, the spin correlation behavior between the top and antitop quarks is sensitive to the CP nature of Higgs states in Yukawa interactions.

**Fig. 2:**
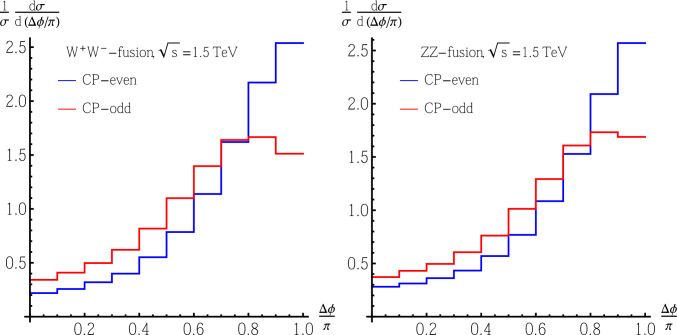
Normalized distributions of the azimuthal angle $\Delta \phi$ [denoted as $\frac{1}{\sigma }\frac{d\sigma }{d(\Delta \phi /\pi )}$] between the charged leptons from $t\bar{t}$ at CLIC with $\sqrt{s}=1.5~\text {TeV}$ for both the $W^+W^-$-fusion (the left plot) and *ZZ*-fusion (the right plot) channels. The blue lines are for a CP-even $Ht\bar{t}$ coupling, while the red lines are for a CP-odd $Ht\bar{t}$ coupling. For such VBF processes, the distributions do not depend on initial beam polarizations

We choose semi-leptonic decay channels $t\left( \bar{t}\right) \rightarrow b\ell ^+\nu \left( \bar{b}\ell ^-\bar{\nu }\right)$ with $\ell =e,\mu$ for both top and antitop quarks. The azimuthal angle between $\ell ^+$ and $\ell ^-$ (denoted as $\Delta \phi$) is a good observable to measure the spin correlations between the top and antitop quarks [87–92]; hence, it is helpful to test the CP nature in the Yukawa sector.^3^ For example, we show the normalized distributions of the azimuthal angle $\Delta \phi$ [denoted as $\frac{1}{\sigma }\frac{d\sigma }{d\Delta \phi }$] between the charged leptons from $t\bar{t}$ at CLIC with $\sqrt{s}=1.5~\text {TeV}$ in Fig. 2 for both the $W^+W^-$- and *ZZ*-fusion channels.

It is clear that the $\Delta \phi$ distributions are different between the cases with CP-even and CP-odd $Ht\bar{t}$ couplings, for both $W^+W^-$- and *ZZ*-fusion channels. As discussed above, VBF production implies the existence of the CP-even component in *H*, and from the $\Delta \phi$ distribution, if we can find evidence of a nonzero $\text {Im}\left( c_t\right)$ (or equivalently the $^1S_0$ type $t\bar{t}$ final state), we can also confirm the CP-odd component in *H*, and hence CP-violation effects. Such a method will prove to be more effective for a heavy scalar *H* mainly containing the CP-odd component, as this is the most different from the CP-conserving case.

### Simulation studies at CLIC

First, we should choose $c_V$ no larger than 0.3, because the global fit for the 125 GeV Higgs boson data implies $c_V\lesssim 0.3$.^4^ The direct LHC search for a heavy scalar *H* decaying to *ZZ* final states sets further limits on $c_V$ if $m_H\lesssim 700~\text {GeV}$ [93], so we choose the LHC-favored region with a benchmark point having $m_H=700~\text {GeV}$ in the following analysis.

In our simulation studies, we choose two cases: CLIC with $\sqrt{s}=1.5~\text {TeV}$ and with $3~\text {TeV}$. The integrated luminosity values are chosen as $L=2.5~(5)~\text {ab}^{-1}$ for $\sqrt{s}=1.5~(3)~\text {TeV}$ [80–82]. Denoting $P_-(P_+)$ as the electron (positron) polarization, we choose the following beam polarization setup: $P_+=0$ for the whole run, meaning the positron beam is always unpolarized; $P_-=-0.8$ for $80\%$ integrated luminosity, and $P_-=+0.8$ for $20\%$ integrated luminosity [80–82].

We consider two VBF processes at CLIC, $W^+W^-$ fusion ($e^+ e^-\rightarrow \nu \overline{\nu }H$) and *ZZ* fusion ($e^+ e^-\rightarrow e^+e^-H$), with the heavy Higgs *H* decaying to a $t\bar{t}$ pair and the top quark and antiquark decaying semileptonically. For the production processes, if the initial electron beam has a nonzero polarization, the cross sections will acquire a factor as [80]

4 $$\begin{aligned}  &   \frac{\sigma _{W^+W^-\rightarrow H,P_-}}{\sigma _{W^+W^-\rightarrow H,\text {unpol.}}}=1-P_-;\quad \quad \frac{\sigma _{ZZ\rightarrow H,P_-}}{\sigma _{ZZ\rightarrow H,\text {unpol.}}} \nonumber \\  &   \quad =\frac{(-\frac{1}{2}+s^2_{\theta _W})^2(1-P_-)+s^4_{\theta _W}(1+P_-)}{(-\frac{1}{2}+s^2_{\theta _W})^2+s^4_{\theta _W}} \nonumber \\    &   \quad =1-0.16P_-. \end{aligned}$$

Here the index “unpol.” means the case without initial beam polarization. Thus, for the case here, $80\%$ of the electron beam with $P_-=-0.8$ and $20\%$ of the electron beam with $P_-=+0.8$ (and keeping $P_+=0$ as above), we denote $\bar{\sigma }$ as the averaged cross section during the whole run, and then we can obtain [80]

5 $$\begin{aligned} \frac{\bar{\sigma }_{W^+W^-\rightarrow H}}{\sigma _{W^+W^-\rightarrow H,\text {unpol.}}}=1.48;\quad \quad \frac{\bar{\sigma }_{ZZ\rightarrow H}}{\sigma _{ZZ\rightarrow H,\text {unpol.}}}=1.08. \end{aligned}$$

The total events number increases a bit in comparison with the unpolarized case.

We assume that the heavy Higgs *H* can decay via only three channels: $H\rightarrow t\overline{t}$, $W^+W^-$, and *ZZ*.^5^ The branching ratio (BR) for the $H\rightarrow t\bar{t}$ decay channel is

6 $$\begin{aligned} \text {BR}_{H\rightarrow t\bar{t}}\equiv \frac{\Gamma _{H\rightarrow t\bar{t}}}{\Gamma _H}=\frac{\Gamma _{H\rightarrow t\bar{t}}}{\Gamma _{H\rightarrow t\bar{t}}+\Gamma _{H\rightarrow W^+W^-}+\Gamma _{H\rightarrow ZZ}},\nonumber \\ \end{aligned}$$

which depends on the couplings $c_V$ and $c_t$. We also have [23]

7 $$\begin{aligned} \Gamma _{H\rightarrow t\bar{t}}= &   \frac{3m_H}{8\pi }\left( \frac{m_t}{v}\right) ^2\left[ \left[ \text {Re}(c_t)\right] ^2\left( 1-\frac{4m^2_t}{m^2_H}\right) ^{\frac{3}{2}}\right. \nonumber \\  &   \left. +\left[ \text {Im}(c_t)\right] ^2 \left( 1-\frac{4m^2_t}{m^2_H}\right) ^{\frac{1}{2}}\right] ,\end{aligned}$$

8 $$\begin{aligned} \Gamma _{H\rightarrow W^+W^-}= &   \frac{m_H^3c_V^2}{16\pi v^2}\sqrt{1-\frac{4m^2_W}{m^2_H}} \left( 1-\frac{4m^2_W}{m^2_H}+\frac{12m^4_W}{m^4_H}\right) ,\nonumber \\ \end{aligned}$$

9 $$\begin{aligned} \Gamma _{H\rightarrow ZZ}= &   \frac{m_H^3c_V^2}{32\pi v^2}\sqrt{1-\frac{4m^2_Z}{m^2_H}}\left( 1-\frac{4m^2_Z}{m^2_H}+\frac{12m^4_Z}{m^4_H}\right) .\nonumber \\ \end{aligned}$$

As we choose $m_H=700~\text {GeV}$ in our simulation studies, we have the total width of *H* as

10 $$\begin{aligned} \Gamma _H=\left( 155c_V^2+26.9\left[ \text {Re}(c_t)\right] ^2+35.5\left[ \text {Im}(c_t)\right] ^2\right) ~\text {GeV}.\nonumber \\ \end{aligned}$$

Typically, we have $|c_t|\sim {\mathcal {O}}(1)$, and $c_V\lesssim 0.3$ based on the results in [25]. Thus, if we choose $c_V=0.3$ and $\left[ \text {Re}(c_t)\right] ^2+1.32\left[ \text {Im}(c_t)\right] ^2=1$ as a benchmark point, numerically we have $\Gamma _H=40.9~\text {GeV}$, which yields a narrow signal peak over the continuum background. The $\text {BR}_{H\rightarrow t\bar{t}}$ has the following numerical dependence on $c_V$ and $c_t$:

11 $$\begin{aligned} \text {Br}_{H\rightarrow t\bar{t}}=\frac{0.174\left[ \text {Re}(c_t)\right] ^2+0.229\left[ \text {Im}(c_t)\right] ^2}{c_V^2+0.174\left[ \text {Re}(c_t)\right] ^2+0.229\left[ \text {Im}(c_t)\right] ^2}. \end{aligned}$$

In Eq. (7,8,9), the term proportional to $\left[ \text {Re}(c_t)\right] ^2$ implies that the partial decay width to $t\bar{t}$ pairs involves a $^3P_0$ state, while the term proportional to $\left[ \text {Im}(c_t)\right] ^2$ implies that the partial decay width to $t\bar{t}$ pairs involves a $^1S_0$ state. The branching ratio of the top quark semileptonic decay is chosen as $21.34\%$, which is the sum of electron and muon channels, as shown in the Particle Data Group (PDG) review [3]. In our simulation studies, we generate the signal and background events at the leading order (LO) using MadGraph5 [94]. We include bremsstrahlung/initial state radiation (ISR) effects through the “isronlyll” option for parton distribution functions (PDFs) [95].

In the $W^+W^-$-fusion channel, the main background is the SM *s*-channel $e^+e^-\rightarrow t\overline{t}$ production because of its high production rate and the fact that the (anti)neutrinos in the final state of the signal cannot be triggered, while other background processes are numerically negligible since their total cross section is two orders of magnitude smaller than the main background $e^+e^-\rightarrow t\overline{t}$, for both $\sqrt{s}=1.5$ and $3~\text {TeV}$. In the *ZZ*-fusion channel, the main background is instead SM $e^+e^-\rightarrow t\overline{t}e^+e^-$ production, which comes from both the VBF production process of $t\overline{t}$ and the $t\overline{t}Z$ associated production process, with the *Z* boson decaying to an electron–positron pair.

**Table 1:** Selection cuts for $W^+W^-$- and *ZZ*-fusion processes at CLIC with $\sqrt{s}=1.5~\text {TeV}$ (the upper two entries) and $\sqrt{s}=3~\text {TeV}$ (the lower two entries)

Process	Selection cuts
$W^+W^-$-fusion ($\sqrt{s}=1.5~\text {TeV}$)	$$\begin{array}{l} n_\ell =2,~n_b=2,~\mid \eta ^{\ell }\mid<3,~\mid \eta ^{b}\mid<3,\\ p_{\text {T}}^{\ell ,b}>10~\hbox {GeV},~p_{\text {T}}^{\hbox {\tiny miss}}<300~\hbox {GeV},~\Delta R_{\ell \ell ,b\ell ,bb}>0.4,\\ m_{bb\ell \ell }<600~\hbox {GeV},~m_{\ell \ell }<350~\hbox {GeV},~m_{\hbox {\tiny inv}}>850~\hbox {GeV}.\\ \end{array}$$
*ZZ*-fusion ($\sqrt{s}=1.5~\text {TeV}$)	$$\begin{array}{l} n_\ell \ge 3,~n_e\ge 1,~n_{\ell ^+}\cdot n_{\ell ^-}>0,~n_b=2,\\ \mid \eta ^{\ell }\mid<3,~\mid \eta ^{b}\mid<3,~\text {max}(\mid \eta ^{\ell }\mid )>2,~\Delta R_{\ell \ell ,b\ell ,bb}>0.4,\\ p_{\textrm{T}}^{\ell ,b}>10~\hbox {GeV},~m_{bb\ell \ell }>350~\hbox {GeV},~m_{\hbox {\tiny inv}}<400~\hbox {GeV}. \end{array}$$
$W^+W^-$-fusion ($\sqrt{s}=3~\text {TeV}$)	$$\begin{array}{l} n_\ell =2,~n_b=2,~\mid \eta ^{\ell }\mid<3,~\mid \eta ^{b}\mid<3,\\ p_{\textrm{T}}^{\ell ,b}>10~\hbox {GeV},~p_{\textrm{T}}^{\hbox {\tiny miss}}<150~\hbox {GeV},~p_{\textrm{T}}^{\ell \ell }<200~\hbox {GeV},~\Delta R_{\ell \ell ,b\ell ,bb}>0.4,\\ m_{bb\ell \ell }<700~\hbox {GeV},~m_{\ell \ell }<450~\hbox {GeV},~m_{\hbox {\tiny inv}}>1900~\hbox {GeV}. \end{array}$$
*ZZ*-fusion ($\sqrt{s}=3~\text {TeV}$)	$$\begin{array}{l} n_\ell \ge 3,~n_e\ge 1,~n_{\ell ^+}\cdot n_{\ell ^-}>0,~n_b=2,\\ \mid \eta ^{\ell }\mid<3,~\mid \eta ^{b}\mid <3,~\text {max}(\mid \eta ^{\ell }\mid )>2,\\ \Delta R_{\ell \ell ,b\ell ,bb}>0.4,~p_{\textrm{T}}^{\ell ,b}>10~\hbox {GeV},~m_{bb\ell \ell }>350~\hbox {GeV}. \end{array}$$

**Table 2:** Averaged cross sections after selection cuts, selection efficiencies, and expected significance for $W^+W^-$- and *ZZ*-fusion processes at CLIC with $\sqrt{s}=1.5~\text {TeV}$ (the upper two entries) and $\sqrt{s}=3~\text {TeV}$ (the lower two entries). Here, $N_{\text {sig}}=\bar{\sigma }_{\text {sig}}L$ and $N_{\text {bkg}}=\bar{\sigma }_{\text {bkg}}L$ are the event rates for signal and background, respectively, after selection cuts, while *L* is the integrated luminosity. The efficiencies for signals are obtained through the cases with pure CP-odd $Ht\bar{t}$-coupling, but numerically we find them nearly independent of the phase in $Ht\bar{t}$-coupling

Process	$\bar{\sigma }_{\text {sig}}$ (fb)	$\epsilon _{\text {sig}}$	$\bar{\sigma }_{\text {bkg}}$ (fb)	$\epsilon _{\text {bkg}}$	$N_{\text {sig}}/\sqrt{N_{\text {bkg}}}$
$W^+W^-$-fusion ($\sqrt{s}=1.5~\text {TeV}$)	$~~~1.38\kappa ~~~$	$~~~64\%~~~$	0.43	$~~~8.9\%~~~$	$~~~106\kappa \sqrt{L/(2.5~\text {ab}^{-1})}~~~$
*ZZ*-fusion ($\sqrt{s}=1.5~\text {TeV}$)	$~~~0.056\kappa ~~~$	$~~~37\%~~~$	$4.8\times 10^{-3}$	$~~~12\%~~~$	$~~~41\kappa \sqrt{L/(2.5~\text {ab}^{-1})}~~~$
$W^+W^-$-fusion ($\sqrt{s}=3~\text {TeV}$)	$~~~4.72\kappa ~~~$	$~~~48\%~~~$	0.042	$~~~3.2\%~~~$	$~~~1.6\times 10^3\kappa \sqrt{L/(5~\text {ab}^{-1})}~~~$
*ZZ*-fusion ($\sqrt{s}=3~\text {TeV}$)	$~~~0.25\kappa ~~~$	$~~~35\%~~~$	0.013	$~~~13\%~~~$	$~~~153\kappa \sqrt{L/(5~\text {ab}^{-1})}~~~$

To reduce the SM backgrounds, we apply the selection cuts in Table 1. For $W^+W^-$-fusion, we are considering the signal process $e^+e^-\rightarrow \nu \bar{\nu }H(\rightarrow t(\rightarrow b\ell ^+\nu )\bar{t}(\rightarrow \bar{b}\ell ^-\bar{\nu }))$, which leads to the final state as , with the missing energy  including at least all the four (anti)neutrinos in the final state. The dominant background process is $e^+e^-\rightarrow t(\rightarrow b\ell ^+\nu )\bar{t}(\rightarrow \bar{b}\ell ^-\bar{\nu }))$, which leads to the same final state as , with the missing energy  including $\nu \bar{\nu }$ together with possible untagged bremsstrahlung/ISR photons. We must tag two *b*-jets, two leptons with opposite signs, together with missing energy. For both $\sqrt{s}=1.5$ and $3~\text {TeV}$, the most important cut is the large invisible invariant mass, denoted as $m_{\text {inv}}$, as part of VBF-tagging. Thus we choose $m_{\text {inv}}>850~(1900)~\text {GeV}$ for $\sqrt{s}=1.5~(3)~\text {TeV}$. Another important cut is the invariant mass for all visible final particles, denoted as $m_{bb\ell \ell }$, which is strongly correlated with the invariant mass of the (anti)top quark pair, $m_{t\bar{t}}$. This is also quite effective for separating the signal and background processes. For the signal process, $b\bar{b}\ell ^+\ell ^-$ must come from the same particle *H*, so we always have $m_{bb\ell \ell }<m_{t\bar{t}}=m_H$, whereas for the background process, $m_{bb\ell \ell }$ is usually larger, since $m_{t\bar{t}}$ is close to $\sqrt{s}$. Thus, we choose $m_{bb\ell \ell }<600~(700)~\text {GeV}$ for $\sqrt{s}=1.5~(3)~\text {TeV}$.

**Table 3:** $\text {N}_{\text {sig}}/\sqrt{\text {N}_{\text {bkg}}}$ for $W^+W^-,ZZ\rightarrow H$ fusion at CLIC, with the largest allowed $c_V=0.3$, fixed $|c_t|=1$, and integrated luminosity $L=2.5~(5)~\text {ab}^{-1}$ at $\sqrt{s}=1.5~(3)~\text {TeV}$

	$W^+W^{-}\text {-fusion}$	ZZ-fusion	$W^+W^{-}\text {-fusion}$	ZZ-fusion
	$(\sqrt{s}=1.5~\text {TeV})$	$(\sqrt{s}=1.5~\text {TeV})$	$(\sqrt{s}=3~\text {TeV})$	$(\sqrt{s}=3~\text {TeV})$
CP-even $Ht\bar{t}$	6.3	2.4	96	9.0
CP-odd $Ht\bar{t}$	6.8	2.6	105	9.9

For *ZZ*-fusion, we consider the signal process $e^+e^-\rightarrow e^+e^-H(\rightarrow t(\rightarrow b\ell ^+\nu )\bar{t}(\rightarrow \bar{b}\ell ^-\bar{\nu }))$, which leads to the final state , with the missing energy  including $\nu \bar{\nu }$ together with a possible untagged forward/backward electron/positron. The dominant background process leads to the same final state. We must tag two *b*-jets and at least three leptons, together with missing energy. In the *ZZ*-fusion process, we have a pair of forward and backward $e^{\pm }$, but we allow one of them to fail detection in order to save more signal events, so we must tag at least one $e^{\pm }$ with $|\eta |>2$ as part of VBF-tagging. We should also tag two other leptons with opposite signs, which come from *t* and $\bar{t}$. Thus, we choose $n_\ell \ge 3,~n_e\ge 1,~n_{\ell ^+}\cdot n_{\ell ^-}>0$, and $\text {max}\left( \left| \eta ^{\ell }\right| \right) >2$. Similar to the $W^+W^-$-fusion case, we also use the $m_{bb\ell \ell }$ cut to separate signal and background processes, since we always have $m_{t\bar{t}}=m_H$ for the signal process whereas in the background process there should be more events with smaller $m_{t\bar{t}}$. Thus, we choose the cut $m_{bb\ell \ell }>350~\text {GeV}$ for both $\sqrt{s}=1.5$ and $3~\text {TeV}$.

After performing these selection cuts, we have the averaged cross sections $\bar{\sigma }_i$ and the corresponding selection efficiencies $\epsilon _i$ for the signal (denoted as the index “sig”) and background (denoted as the index “bkg”) processes in Table 2, together with the discovery potential as a function of the machine luminosity *L*. We use “averaged” here because the cross section for a process will change with the electron polarization $P_-$, and thus it is not a constant during the whole run. For the rates in the table, we know that the signal production cross sections are proportional to the parameter

12 $$\begin{aligned} \kappa\equiv &   \frac{c_V^2\left( 0.174\left[ \text {Re}(c_t)\right] ^2+0.229\left[ \text {Im}(c_t)\right] ^2\right) }{c_V^2+0.174\left[ \text {Re}(c_t)\right] ^2+0.229\left[ \text {Im}(c_t)\right] ^2}\nonumber \\  = &   c_V^2\text {Br}_{H\rightarrow t\bar{t}}. \end{aligned}$$

Since $c_V\lesssim 0.3$ [25], if we fix $|c_t|=1$, the largest allowed number for this parameter should be $\kappa _{\text {max}}^+=0.059$ for a CP-even $Ht\bar{t}$ coupling and $\kappa _{\text {max}}^-=0.065$ a for CP-odd $Ht\bar{t}$ coupling. If we choose the typical integrated luminosity $L=2.5~(5)~\text {ab}^{-1}$ at $\sqrt{s}=1.5~(3)~\text {TeV}$, and the largest allowed $c_V=0.3$, we list the numbers of $N_{\text {sig}}/\sqrt{N_{\text {bkg}}}$ for pure CP-even and CP-odd $Ht\bar{t}$-coupling in Table 3.

Based on the results in Table 3, we find that both $W^+W^-$- and *ZZ*-fusion channels at CLIC with $\sqrt{s}=3~\text {TeV}$, and $W^+W^-$-fusion channel at CLIC with $\sqrt{s}=1.5~\text {TeV}$, can be discovered quite promptly with the significance larger than $5\sigma$ for both CP-even and CP-odd $Ht\bar{t}$ couplings, so that we have the basis to further analyze the final state $\Delta \phi$ distributions to probe the CP nature of the *H* state. The *ZZ*-fusion channel at CLIC with $\sqrt{s}=1.5~\text {TeV}$ has lower significance, due to its smaller number of events.

### Analysis and results

**Fig. 3:**
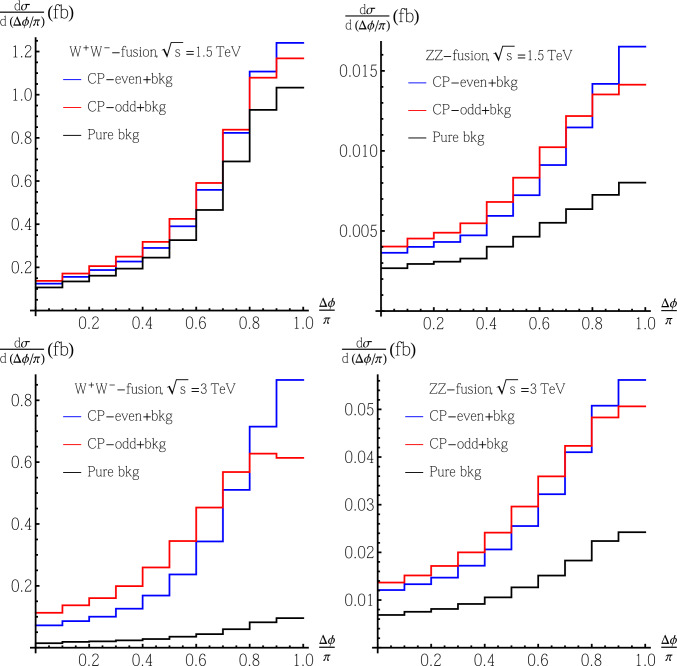
Differential cross sections in the azimuthal angle $\Delta \phi$ [denoted as $\frac{d\sigma }{d(\Delta \phi /\pi )}$] between the charged leptons from $t\bar{t}$ at CLIC including both signal and background events with fixed $|c_t|=1$ and $c_V=0.3$: the upper plots are for the case with $\sqrt{s}=1.5~\text {TeV}$ and the lower plots are for the case with $\sqrt{s}=3~\text {TeV}$, while the left plots are for the $W^+W^-$-fusion process and the right plots are for the *ZZ*-fusion process. The blue lines are for a CP-even $Ht\bar{t}$ coupling together with background events while the red lines are for a CP-odd $Ht\bar{t}$ coupling together with background events, while we also show the distribution for pure background events as black lines

For all four cases (both $W^+W^-$- and *ZZ*-fusion, with $\sqrt{s}=1.5~\text {TeV}$ and $3~\text {TeV}$), assuming $|c_t|=1$ and $c_V=0.3$ (which is the largest allowed $c_V$, corresponding to the largest allowed $\kappa$ under fixed $c_t$), we show the $\Delta \phi$ distribution in Fig. 3, including both signal and background events.

We also add the $\Delta \phi$ distribution for pure background events for comparison. The plots clearly show differences between the two CP hypotheses in the $\Delta \phi$ distribution even after adding the background events.

We then define the asymmetry between the events with large ($\Delta \phi >\pi /2$) and small ($\Delta \phi <\pi /2$) azimuthal angle as

13 $$\begin{aligned} {A}\equiv \frac{N_{\Delta \phi>\pi /2}-N_{\Delta \phi<\pi /2}}{N_{\Delta \phi >\pi /2}+N_{\Delta \phi <\pi /2}}=\frac{N_+-N_-}{N_++N_-}, \end{aligned}$$

which is sensitive to the CP nature of the $Ht\bar{t}$ coupling. Its statistical uncertainty can be calculated through [25]

14 $$\begin{aligned} \sigma _{{A}}=\sqrt{\frac{4N_+N_-}{N^3}} \end{aligned}$$

if $N_{\pm }\gg 1$, and $N=N_++N_-$ is the total number of events. In our analysis, for all cases, we must consider the signal and background events together, since they become indistinguishable experimentally even after our selection cuts. Thus, $N_{\pm }$ contains both signal and background events.

**Fig. 4:**
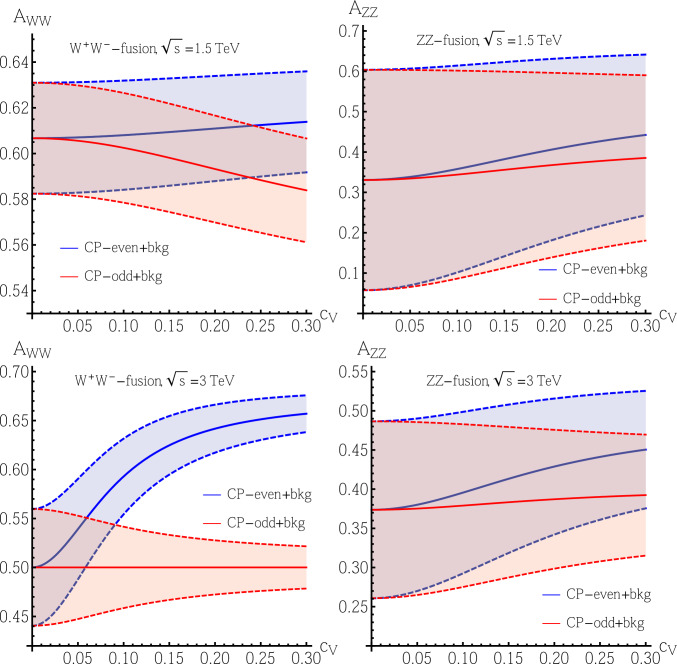
Asymmetries *A* including both signal and background events: the upper plots are for the case with $\sqrt{s}=1.5~\text {TeV}$ and the lower plots are for the case with $\sqrt{s}=3~\text {TeV}$; the left plots are for the $W^+W^-$-fusion process and the right plots are for the *ZZ*-fusion process. We fix $[\text {Re}(c_t)]^2+1.32[\text {Im}(c_t)]^2=1$ for all processes. The blue lines are for ${A}^+$ including background events while the red lines are for ${A}^-$ including background events. The solid lines are the central values ${A}^{\pm }$ while the dashed lines are ${A}^{\pm }\pm \sigma _{{A}^{\pm }}$, where $\sigma _{{A}^{\pm }}$ are the $1\sigma$ uncertainties for ${A}^{\pm }$, with the integrated luminosity $L=2.5~(5)~\text {ab}^{-1}$ for $\sqrt{s}=1.5~(3)~\text {TeV}$

We calculate such an asymmetry *A* for each process (denoted through the sub-indices $W^+W^-$ and *ZZ* in the plots) for both pure CP-even $(+)$ and pure CP-odd $(-)$ $Ht\bar{t}$ couplings; hence, we use ${A}^{\pm }$ (in the subsequent text), together with their $\pm 1\sigma$ uncertainties $\sigma _{{A}^{\pm }}$ assuming as integrated luminosity $L=2.5~(5)~\text {ab}^{-1}$ for $\sqrt{s}=1.5~(3)~\text {TeV}$, and show the results in Fig. 4. In the calculation for *A* in Fig. 4, we fix $[\text {Re}(c_t)]^2+1.32[\text {Im}(c_t)]^2=1$ as a benchmark point, so that the parameter $\kappa$ defined in Eq. (12) becomes $\kappa =0.174c_V^2/(c_V^2+0.174)$, and thus the total cross sections do not depend on $\arg (c_t)\equiv \arctan [\text {Im}(c_t)/\text {Re}(c_t)]$.

For the case with CP-mixing $Ht\bar{t}$ coupling, the asymmetry *A* will be located between blue and red lines. In our method, a nonzero $\text {Im}(c_t)$ is enough to probe CP-violation; thus we choose a pure CP-odd $Ht\bar{t}$ coupling to find the largest deviation from the CP-conserving case (corresponding to CP-even $Ht\bar{t}$ coupling). For given experimental conditions and model parameters, the number of standard deviations

15 $$\begin{aligned} s_0=\frac{|{A}-{A}^+|}{\sigma _{{A}^+}} \end{aligned}$$

away from the CP-conserving case measures the significance for discovering CP-violation, so that for a pure CP-odd $Ht\bar{t}$ coupling, we have ${A}={A}^-$, which will give us the largest significance for CP-violation.

From the right plots in Fig. 4, it is clear that in the *ZZ*-fusion channel, it is difficult to distinguish between a CP-even and CP-odd $Ht\bar{t}$ coupling. Even for the largest allowed value $c_V=0.3$, we still have $s_0<1$, meaning that ${A}^-$ is quite close to ${A}^+$. This is mainly because of the small cross section and hence event number of the *ZZ*-fusion process, in turn adversely affecting the error. Thus, for the *ZZ*-fusion channel, we do not need further analysis. For $W^+W^-$-fusion with $\sqrt{s}=1.5~\text {TeV}$, and upon choosing the largest allowed $c_V=0.3$, $s_0\simeq 1.4$, meaning quite a slight deviation but still not strong enough evidence to discover CP-violation. This is because at the $\sqrt{s}=1.5~\text {TeV}$ CLIC, there is still a large $t\bar{t}$ background which cannot be effectively reduced, i.e., $\sigma _{\text {bkg}}\gg \sigma _{\text {sig}}$. The large background erases the difference between ${A}^+$ and ${A}^-$, and thus only a $1.4\sigma$ deviation is left even for $c_V=0.3$. We do not further analyze this case then. For $W^+W^-$-fusion with $\sqrt{s}=3~\text {TeV}$, from the lower-left plot in Fig. 4, if $c_V\gtrsim 0.12$, we have $s_0\gtrsim 3$, meaning that ${A}^-$ and ${A}^+$ are significantly different in this case.

Therefore, we further analyze the asymmetry in the $W^+W^-$-fusion process at CLIC with $\sqrt{s}=3~\text {TeV}$. Experimentally, the two useful observables are the total cross section $\sigma _{\text {tot}}=\sigma _{\text {sig}}+\sigma _{\text {bkg}}$ and the asymmetry *A* in the $\Delta \phi$ distribution. Note that $\sigma _{\text {tot}}$ depends only on the parameter $\kappa$, while *A* depends on both $\kappa$ and $\xi \equiv \arg (c_t)$. Numerically, we have

16 $$\begin{aligned} \sigma _{\text {tot}}= &   \sigma _{\text {sig}}+\sigma _{\text {bkg}}=(4.72\kappa +0.042)~\text {fb}, \end{aligned}$$

17 $$\begin{aligned} {A}= &   \frac{1}{\sigma _{\text {tot}}}\left( {A}_{\text {bkg}}\sigma _{\text {bkg}}+\frac{c^2_{\xi } {A}^++1.32s^2_{\xi }{A}^-}{c^2_{\xi }+1.32s^2_{\xi }}\sigma _{\text {sig}}\right) \nonumber \\= &   \frac{1}{\kappa +8.95\times 10^{-3}}\nonumber \\    &   \left( 4.47\times 10^{-3}+\frac{0.681c^2_{\xi }+0.659s^2_{\xi }}{c^2_{\xi }+1.32s^2_{\xi }}\kappa \right) . \end{aligned}$$

**Fig. 5:**
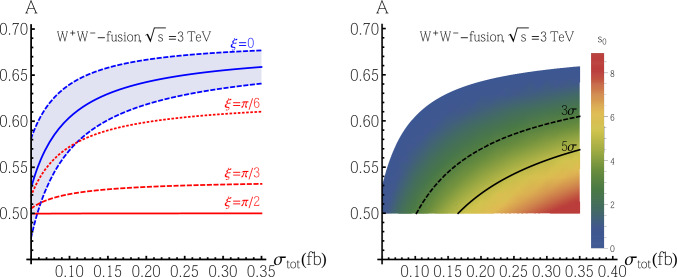
In the left plot: we show the expected correlation between the asymmetry *A* and total cross section $\sigma _{\text {tot}}$ for different $\xi \equiv \arg (c_t)$: the blue line with $\xi =0$ means pure CP-even $Ht\bar{t}$ coupling corresponding to the CP-conserving case (together with its $\pm 1\sigma$ uncertainty) while the three red lines with $\xi =\pi /6,\pi /3,\pi /2$ correspond to CP-violation cases. In the right plot: we show the standard deviation away from the CP-conserving case (denoted as $s_0=|{A}-{A}^+|/\sigma _{A^+}$, meaning the discovery potential for CP-violation) in the ${A}-\sigma _{\text {tot}}$ plane, together with the $3\sigma$ (dashed black line) and $5\sigma$ (solid black line) evidence and discovery boundaries, respectively

With the typical luminosity $L=5~\text {ab}^{-1}$, the relative uncertainty of the total cross section $\sigma _{\text {tot}}$ is estimated through $\delta \sigma _{\text {tot}}/\sigma _{\text {tot}}=1/\sqrt{N_{\text {sig}}+N_{\text {bkg}}}\sim {\mathcal {O}}(10^{-2})$^6^, which is ignorable compared with the relative uncertainty of *A*. In Fig. 5, we show the correlation between asymmetry *A* and total cross section $\sigma _{\text {tot}}$ for different $\xi$ in the left plot, together with the standard deviation away from the CP-conserving case (denoted as $s_0=|{A}-{A}^+|/\sigma _{A^+}$, meaning the discovery potential for CP-violation) for different observed asymmetry *A* and total cross section $\sigma _{\text {tot}}$ values in the right plot.

If $\sigma _{\text {tot}}\gtrsim 0.10~\text {fb}$ (corresponding to $\kappa \gtrsim 0.013$, or $c_V\gtrsim 0.12$), a pure CP-odd $Ht\bar{t}$ coupling is expected to be evidenced at the $3\sigma$ level, while if $\sigma _{\text {tot}}\gtrsim 0.17~\text {fb}$ (corresponding to $\kappa \gtrsim 0.026$, or $c_V\gtrsim 0.17$), a pure CP-odd $Ht\bar{t}$ coupling is expected to be discovered at $5\sigma$ level. For the largest allowed $c_V\simeq 0.3$ (corresponding to $\kappa \simeq 0.065$, or $\sigma _{\text {tot}}\simeq 0.35~\text {fb}$), a pure CP-odd $Ht\bar{t}$ coupling corresponding to $\xi =\pi /2$ is expected to be discovered at the $8.8\sigma$ level, while the $3(5)\sigma$ evidence (discovery) boundary corresponds to $\xi \simeq 0.18\pi (0.25\pi )$. Here, we assume $|c_t|=1$ for simplicity if necessary.

## Implication for the 2HDM with CP-violation

### Model setup

We choose the 2HDM with CP-violation [23] as a test model in this section. Mainly following the conventions in [25, 96], the Lagrangian in the scalar sector is

18 $$\begin{aligned} {\mathcal {L}}\supset \mathop {\sum }_{i=1,2} \, \left( D_{\mu }\phi _i \right) ^{\dag }\, \left( D^{\mu }\phi _i \right) -V\left( \phi _1,\phi _2\right) , \end{aligned}$$

where $\phi _1=\left( \varphi _1^+,\frac{1}{\sqrt{2}}(v_1+\eta _1+\text {i}\chi _1)\right) ^T$ and $\phi _2= \left( \varphi _2^+,\frac{1}{\sqrt{2}}(v_2+\eta _2+\text {i}\chi _2)\right) ^T$ are two $\text {SU}(2)$ doublets. The vacuum expectation values (VEVs) $v_{1,2}$ satisfy the relation $v=\sqrt{|v_1|^2+|v_2|^2}=246~\text {GeV}$. We also define $t_{\beta }\equiv |v_2/v_1|$ as usual. The scalar potential is given by

19 $$\begin{aligned} V\left( \phi _1,\phi _2\right)= &   -\frac{1}{2}\left[ m_1^2\phi _1^{\dag }\phi _1+m_2^2\phi _2^{\dag }\phi _2\right. \nonumber \\    &   \left. +\left( m^2_{12} \phi _1^{\dag }\phi _2+\text {H.c.}\right) \right] \nonumber \\  &   +\frac{1}{2}\left[ \lambda _1\left( \phi _1^{\dag }\phi _1\right) ^2+\lambda _2\left( \phi _2^{\dag }\phi _2\right) ^2\right] \nonumber \\  &   +\lambda _3\left( \phi _1^{\dag }\phi _1\right) \left( \phi _2^{\dag }\phi _2\right) +\lambda _4\left( \phi _1^{\dag }\phi _2\right) \left( \phi _2^{\dag }\phi _1\right) \nonumber \\  &   +\left[ \frac{\lambda _5}{2}\left( \phi _1^{\dag }\phi _2\right) ^2+\text {H.c.}\right] , \end{aligned}$$

where we assumed a softly broken $Z_2$ symmetry^7^ to avoid the possible tree-level flavor-changing neutral current (FCNC) interactions. Here, $m_{12}^2$, $\lambda _5$, and $v_2/v_1$ can be complex parameters, and we can always perform a field rotation to make at least one of them real. We choose $v_2/v_1$ to be real^8^, and thus the vacuum conditions lead us to the relation [25, 96]

20 $$\begin{aligned} \text {Im}\left( m^2_{12}\right) =v_1v_2\text {Im}\left( \lambda _5\right) . \end{aligned}$$

**Fig. 6:**
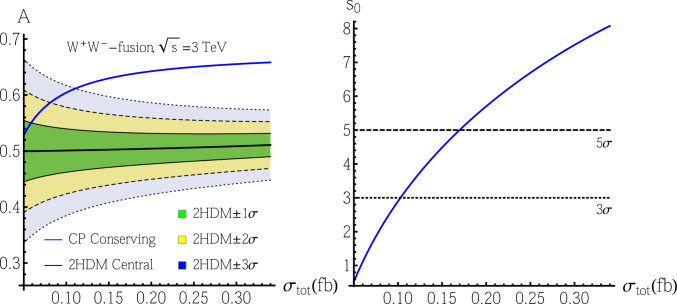
In the left plot: we show the predicted asymmetry *A* versus the total cross section $\sigma _{\text {tot}}$ in the chosen 2HDM with $\pm 1\sigma$ (green), $\pm 2\sigma$ (yellow), and $\pm 3\sigma$ (blue) uncertainties. The thick black line shows the central value of the 2HDM prediction. We also show the CP-conserving prediction as the thick blue line as a comparison. In the right plot: we show the discovery potential of CP-violation versus the total cross section $\sigma _{\text {tot}}$ if an asymmetry *A* equal to the 2HDM prediction is observed

If both sides in the equation above are nonzero, there will be CP-violation in the scalar sector. The Goldstone modes $G^+$ and $G^0$ can be recovered through a diagonalization procedure as

21 $$\begin{aligned}  &   \left( \begin{array}{c}G^+\\ H^+\end{array}\right) =\left( \begin{array}{cc}c_{\beta }& s_{\beta }\\ -s_{\beta }& c_{\beta }\end{array}\right) \left( \begin{array}{c}\varphi _1^+\\ \varphi _2^+\end{array}\right) ,\quad \text {and}\nonumber \\  &   \left( \begin{array}{c}G^0\\ A^0\end{array}\right) =\left( \begin{array}{cc}c_{\beta }& s_{\beta }\\ -s_{\beta }& c_{\beta }\end{array}\right) \left( \begin{array}{c}\chi _1\\ \chi _2\end{array}\right) . \end{aligned}$$

If there is no CP-violation, $A^0$ should be a pure pseudoscalar, while in the CP-violation scenario, $A^0$ must further mix with $\eta _{1,2}$ to obtain the neutral mass eigenstates as

22 $$\begin{aligned} \left( \begin{array}{c}H_1\\ H_2\\ H_3\end{array}\right) =R\left( \begin{array}{c}\eta _1\\ \eta _2\\ A^0\end{array}\right) . \end{aligned}$$

The mixing matrix *R* is parameterized following the convention in [25] as

23 $$\begin{aligned} R=\left( \begin{array}{ccc}1& & \\   & c_{\alpha _3}& s_{\alpha _3}\\   & -s_{\alpha _3}& c_{\alpha _3}\end{array} \right) \left( \begin{array}{ccc}c_{\alpha _2}& & s_{\alpha _2}\\ & 1&  \\ -s_{\alpha _2}& & c_{\alpha _2}\end{array}\right) \left( \begin{array}{ccc}c_{\beta +\alpha _1}& s_{\beta +\alpha _1}& \\ -s_{\beta +\alpha _1}& c_{\beta +\alpha _1}& \\ & & 1\end{array}\right) .\nonumber \\ \end{aligned}$$

With this convention, if $\alpha _{1,2}\rightarrow 0$, $H_1$ becomes the SM Higgs boson. Here, $\alpha _2$ is an important parameter because it measures the CP-violation mixing corresponding to the SM-like Higgs boson $H_1$. In the Yukawa sector, a fermion bilinear can couple to only one scalar doublet due to the $Z_2$ symmetry. Denoting $Q_L\equiv (u,d)_L^T$ and $L_L\equiv (\nu ,\ell )_L^T$, we always assume that $\bar{Q}_Lu_R$ couples to $\phi _2$, and thus the four types of Yukawa interactions are

24 $$\begin{aligned} {\mathcal {L}}\supset \left\{ \begin{array}{cl} -Y_U\bar{Q}_L\tilde{\phi }_2U_R-Y_D\bar{Q}_L\phi _2D_R-Y_{\ell }\bar{L}_L\phi _2\ell _R\\ \quad +\text {H.c.},\quad ~(\text {Type I}),\\ -Y_U\bar{Q}_L\tilde{\phi }_2U_R-Y_D\bar{Q}_L\phi _1D_R-Y_{\ell }\bar{L}_L\phi _1\ell _R\\ \quad +\text {H.c.},\quad ~(\text {Type II}),\\ -Y_U\bar{Q}_L\tilde{\phi }_2U_R-Y_D\bar{Q}_L\phi _2D_R-Y_{\ell }\bar{L}_L\phi _1\ell _R\\ \quad +\text {H.c.},\quad ~(\text {Type III}),\\ -Y_U\bar{Q}_L\tilde{\phi }_2U_R-Y_D\bar{Q}_L\phi _1D_R-Y_{\ell }\bar{L}_L\phi _2\ell _R\\ \quad +\text {H.c.},\quad ~(\text {Type IV}). \end{array}\right. \end{aligned}$$

Following our analysis in [25], the type I and IV models face very stringent electron EDM constraints, and thus the CP-violation mixings are limited to ${\mathcal {O}}\left( 10^{-3}\right)$. However, in type II and III models, a possible cancellation between different contributions to the electron EDM leads to a much weaker constraint on the CP-violation mixing $\alpha _2$ [25, 29, 32–34, 42, 97–103]. As shown in [25], in the type II model, $|\alpha _2|\lesssim 0.1$, mainly due to the neutron EDM constraint, while in the type III model, $|\alpha _2|\lesssim 0.3$, mainly due to the global-fit on LHC Higgs data.^9^ The cancellation appears around $t_{\beta }\simeq 1$,^10^ depending weakly on $m_{2,3}$ and $\alpha _2$, so we choose the type III model as an example. We consider the case for which $H_{2,3}$ have a large mass splitting and $H=H_2$ is dominated by the pseudoscalar component; thus, $\alpha _3\sim \pi /2$, and we have the relation

25 $$\begin{aligned} t_{\alpha _3}=\frac{\left( m_3^2-m^2_2\right) +\sqrt{\left( m^2_3-m^2_2\right) ^2s^2_{2\beta +\alpha _1}-4 \left( m^2_3-m_1^2\right) \left( m^2_2-m^2_1\right) s^2_{\alpha _2} c^2_{2\beta +\alpha _1}}}{2\left( m_2^2-m^2_1\right) s_{\alpha _2}c_{2\beta +\alpha _1}}. \end{aligned}$$

In the type III 2HDM, when $\alpha _1\simeq 0$, $\alpha _3\simeq \pi /2$ and $t_{\beta }\simeq 1$, the coefficients in Eq. (3) are reduced to

26 $$\begin{aligned} c_V\simeq -s_{\alpha _2},\quad \quad \text {and}\quad \quad c_t\simeq -s_{\alpha _2}-\text {i}c_{\alpha _2}=-\text {e}^{\text {i}\left( \pi /2-\alpha _2\right) }.\nonumber \\ \end{aligned}$$

Thus, $\alpha _2$ is a key parameter measuring CP-violation in the (pseudo)scalar sector.

### Implications of CP-violation in the 2HDM

If we choose a scenario with the aforementioned cancellations in the electron EDM which allows larger CP-violation angle $\alpha _2$, we have the expected correlation between the asymmetry *A* and the total cross section $\sigma _{\text {tot}}$, as discussed in Sect. 2.3. Both *A* and $\sigma _{\text {tot}}$ depend only on the parameter $\alpha _2$ for a given $m_H$ (and hence the electron EDM cancellation condition will fix $t_{\beta }\simeq 1$). We numerically obtain the dependence through Eqs. (16) and (17). We confirm the CP-even component of *H* through the nonzero VBF signal cross section, which significantly affects $\sigma _{\text {tot}}$, as above. We confirm the CP-odd component of *H* by measuring the asymmetry *A* and comparing the result with the CP-conserving case. In this scenario, the $Ht\bar{t}$ coupling is dominated by the CP-odd component and thus the expected asymmetry *A* should be close to the case with pure CP-odd $Ht\bar{t}$ coupling. The $H\rightarrow Zh$ decay channel is negligible here.

In this section, we choose the $W^+W^-$-fusion channel at CLIC with $\sqrt{s}=3~\text {TeV}$ and $5~\text {ab}^{-1}$ luminosity, as above. In the left plot of Fig. 6, we show the predicted asymmetry *A* depending on the total cross section $\sigma _{\text {tot}}$ in this scenario of a 2HDM with $\pm 1\sigma ,\pm 2\sigma ,\pm 3\sigma$ uncertainties, together with the prediction from the CP-conserving case for comparison. In the right plot of Fig. 6, we show the discovery potential of CP-violation depending on the total cross section $\sigma _{\text {tot}}$ if an asymmetry *A* equaling the 2HDM prediction is observed.

For the left plot, if an observed $(\sigma _{\text {tot}},{A})$ point is located outside the yellow (blue) boundaries, it will mean that the 2HDM scenario we discuss here is excluded at a $95\% (99.7\%)$ confidence level (C.L.), and thus this 2HDM scenario is disfavored. If an asymmetry *A* equal to the prediction by this 2HDM scenario is observed, meaning this 2HDM scenario is favored, we show the discovery potential of CP-violation in the right plot. We can discover CP-violation at $3(5)\sigma$ level if $\sigma _{\text {tot}}\gtrsim 0.10(0.17)~\text {fb}$, corresponding to $|\alpha _2|\gtrsim 0.12(0.18)$. Finally, for the largest allowed $|\alpha _2|\simeq 0.3$, corresponding to $\sigma _{\text {tot}}\simeq 0.34~\text {fb}$, we can discover CP-violation at the $8.1\sigma$ level.

## Conclusions and discussion

In this paper, we propose testing CP-violation in a (pseudo)scalar sector characterized by a heavy scalar boson *H* with couplings to the weak gauge bosons and a complex coupling to the top quark. We have studied the physics potential of an electron–positron collider at $\sqrt{s}=1.5$ and 3 TeV, such as CLIC, with the beam polarization setup as follows: $P_+=0$ in the whole run, while $P_-=-0.8(+0.8)$ with $80\%(20\%)$ integrated luminosity. At such high energies, the production of a heavy scalar boson is dominated by $W^+W^-$- and *ZZ*-fusion. We choose the process in which the heavy scalar *H* is produced through the VBF channel, and decays to a $t\bar{t}$ pair. In our method, the CP-even component of *H* is confirmed through the *HVV* coupling, while the CP-odd component of *H* should be confirmed through the CP-odd $Ht\bar{t}$ coupling. The CP nature of $Ht\bar{t}$ coupling is tested through the spin correlation between *t* and $\bar{t}$, which is sensitive to the distribution of the azimuthal angle between the leptons decaying from *t* and $\bar{t}$ quarks.

In our study, we found that the *ZZ*-fusion channel suffers from the SM background and cannot provide large enough significance to see the effect of CP-violation even under the most favorable scenario of CP-violation at $\sqrt{s}=1.5$ or 3 TeV. In contrast, the $W^+W^-$-fusion channel provides a small yet visible separation of pure CP-even and CP-odd $Ht\bar{t}$ coupling at $\sqrt{s}=1.5$ TeV, and the significant difference (more than $5\sigma$) between the CP-even and CP-odd $Ht\bar{t}$ coupling can be seen at $\sqrt{s}=3$ TeV CLIC with $5~\text {ab}^{-1}$ luminosity, under a favorable scenario of CP-violation. The physics potential is summarized in Fig. 5, where one can see that a pure CP-odd $Ht {\bar{t}}$ coupling can be discovered at $5\sigma$ level for $\sigma _{\textrm{tot}}\simeq 0.17$ fb (corresponding to $c_V\simeq 0.17$ if assuming $|c_t|=1$), and it can be stretched to $8.8\sigma$ for $\sigma _{\textrm{tot}}\simeq 0.35$ fb (corresponding to the largest allowed $c_V\simeq 0.3$ if assuming $|c_t|=1$).

Implications for the 2HDM with CP-violation in the Higgs sector were also studied. The type III model affords a fairly large CP-violating angle $\alpha _2$, such that this scenario can be analyzed similarly to what we did for the model-independent approach. The results are summarized in Fig. 6. Eventually, we showed that at $\sqrt{s}=3$ TeV CLIC with $5~\text {ab}^{-1}$ luminosity, the 2HDM type III with a favorable CP-violating setup can be discovered at $5\sigma$ level when $\sigma _{\textrm{tot}}\simeq 0.17$ fb (corresponding to $|\alpha _2|\simeq 0.18$), and it can be stretched to $8.1\sigma$ when $\sigma _{\textrm{tot}}\simeq 0.34$ fb (corresponding to the largest allowed $|\alpha _2|\simeq 0.3$).

In short, an electron–positron collider operating in the multi-TeV energy range, such as CLIC, is a useful apparatus to study CP-violation effects in the (pseudo)scalar Higgs sector by using VBF production (through the charged current channel) of a heavy Higgs state decaying into a $t{\bar{t}}$ pair, in turn yielding two (prompt) leptons. We have come to this conclusion by performing an estimated Monte Carlo (MC) analysis, albeit limited to the parton level. However, we are confident that our results can be replicated at the full detector level, given that they are driven by inclusive and exclusive observables solely exploiting electron and muon kinematics.

## Data Availability

This manuscript has no associated data. [Authors’ comment: This is a theoretical research paper without obtaining data from the real world or analyzing them.]

## Footnotes

1 For this topic, see also similar phenomenological studies in a variety of alternative BSM scenarios [26–46].

2 In this paper, we use $s_{\theta }\equiv \sin \theta$, $c_{\theta }\equiv \cos \theta$, and $t_{\theta }\equiv \tan \theta$ for any angle $\theta$ to simplify notation.

3 A similar behavior appears in $t\bar{t}h$-associated production, in which $\Delta \phi$ is one of the best observables to probe CP-violation in $ht\bar{t}$ interaction [25, 38, 39, 90, 91].

4 Or else the couplings between the 125 GeV Higgs boson and massive gauge bosons will be too small to satisfy the LHC data; see more detailed analysis in [25].

5 Here we do not consider the *ZHh* coupling for simplification.

6 This estimation comes only from our signal process $WW\rightarrow H\rightarrow t(\rightarrow b\ell ^+\nu )\bar{t}(\rightarrow \bar{b}\ell ^-\bar{\nu })$. With the help of other decay channels of *H* and $t(\bar{t})$, we will obtain a better estimation on the uncertainty of $\sigma _{\text {tot}}$.

7 Under the $Z_2$ transformation, $\phi _1\rightarrow \phi _1$ and $\phi _2\rightarrow -\phi _2$ and in Eq. (19) only the $m_{12}^2$-term breaks this symmetry.

8 Indeed, we can then make sure both $v_1$ and $v_2$ are real through gauge transformations.

9 As shown in [25], the difference comes from the neutron EDM calculation. An accidental partial cancellation in the type III model makes the constraints from the neutron EDM much weaker than in the type II model.

10 The updated EDM data through $\text {HfF}^+$ measurement [11] does not change this result. As shown in [97, 103], if we consider merely the constraints from electron EDM, a large $t_{\beta }\sim {\mathcal {O}}(10)$ is also possible to lead to an accidental cancellation so that a large CP-violation phase is allowed. However, this parameter region will be strictly constrained by the EDMs of diamagnetic atoms (such as $^{199}\text {Hg}$). This was discussed briefly in [25], and more details will appear in a forthcoming paper. Thus, in this paper, we only choose the benchmark point $t_{\beta }\simeq 1$.

## Code Availability Statement

This manuscript has no associated code/software. [Authors’ comment: We used public software during the study as mentioned in the text. Anybody can reproduce our calculation using the public software.]
